# Troubleshooting a Nonresponder: Guidance for the Strength and Conditioning Coach

**DOI:** 10.3390/sports9060083

**Published:** 2021-06-05

**Authors:** Benjamin H. Gleason, William G. Hornsby, Dylan G. Suarez, Matthew A. Nein, Michael H. Stone

**Affiliations:** 1 Department of Kinesiology, Louisiana Tech University, Ruston, LA 71272, USA; 2 College of Physical Activity and Sport Sciences, West Virginia University, Morgantown, WV 26505, USA; william.hornsby@mail.wvu.edu; 3 Center of Excellence for Sport Science and Coach Education, Department of Sport, Exercise, Recreation, & Kinesiology, East Tennessee State University, Johnson City, TN 37614, USA; dylangsuarez@gmail.com (D.G.S.); stonem@etsu.edu (M.H.S.); 4 Department of Athletics, Salisbury University, Salisbury, MD 21801, USA; manein@salisbury.edu

**Keywords:** coach evaluation, sport performance, high performance

## Abstract

Ideally an athlete would continue to improve performance indefinitely over time, however improvement slows as the athlete approaches their genetic limits. Measuring performance is complex—performance may be temporarily depressed following aggressive training for multiple reasons, physiological and psychosocial. This reality may be vexing to the strength and conditioning coach, who, as a service provider, must answer to sport coaches about an athlete’s progress. Recently an evaluation mechanism for strength and conditioning coaches was proposed, in part to help coaches establish their effectiveness within the organization. Without formal guidance and realistic expectations, if an athlete is not bigger, leaner, stronger, etc. as a result of training within a specified timeframe, blame is often placed upon the strength and conditioning coach. The purpose of this article is to explore possible causes of what may be perceived as athlete non-responses to training and to provide guidance for the coach on how to handle those issues within their domain. A process of investigation is recommended, along with resources to assist coaches as they consider a broad range of issues, including enhancing existing testing methods, improving athlete behaviors, and adjusting processes designed to bring about performance improvement.

## 1. Introduction

Substantial performance enhancement is common early in an athlete’s career, and may be expected as a result of training. As athletes progress from novice toward the advanced or elite levels, rate of performance improvement slows [1,2,3]. In addition, the rate of change of training-related physiological adaptations varies from person to person due to genetic, training methodology, psychosocial, and environmental factors [2,4,5,6,7], making performance difficult to predict and improvements challenging to demonstrate at times [8]. Furthermore, the genetic limitations on the “window of adaptation” are lower in some athletes compared to others, thus those athletes with a smaller window will show asymptotic changes in performance earlier than those with a large window [7,9]. In addition, competitive performances of elite and professional athletes have shown a range of variability, depending on the sport. For instance, Malcata and Hopkins [10] showed intra-athlete performances varied from 0.15% to 53%, with subjectively-judged sports demonstrating less-stable performances.

It is necessary to identify what would characterize a downward trend in performance for an individual athlete compared to acceptable variation. Based upon several decades of discussion in the literature, overreaching has been described as: an accumulation of training and/or non-training stress resulting in a relatively short-term decrement in performance capacity with accompanying physiological and psychological signs and symptoms of maladaptation in which restoration of performance capacity may take from several days to weeks [11,12]. Overtraining has been described as a similar phenomenon that can require many weeks to months for performance ability restoration [11,12]. Further discussion split overreaching into two categories: functional (FOR) and nonfunctional (NFOR) overreaching. FOR is a process resulting in temporarily depressed performance (days to weeks) that does rebound often to higher levels (supercompensation) with recovery/rest—and therefore is considered acceptable and often beneficial [13]. A more severe phenomenon, NFOR has been classified as a longer-term depression of performance that typically requires a recovery of several weeks to months [14]. In contrast, overtraining syndrome (OTS) is a maladaptive condition in which a long-term depression of performance is observed [14]. OTS has been demonstrated to result in neurological, endocrine, immune, and mood disturbances for months; the condition is challenging to diagnose because of the complexity in pathophysiology and the arbitrary nature of definitions used [15]. OTS may require a detailed medical evaluation in order to diagnose effectively [16], and there are many potential symptoms—both sympathetic and parasympathetic in nature [12]. It is important to note that training monotony has also been implicated in maladaptation to training [12], which presents a promising area for coaches and sport support specialists to explore using monitoring methods to quantify training loads and establish a more informed decision-making process in which training goals are balanced with the reality of athlete status [17,18,19,20].

For application by the strength and conditioning (SC) coach, the difference between variations of overreaching and overtraining is simply performance rebounding at least to prior levels at the completion of the training phase. In principle, the outcome of such a process where performance has rebounded is likely to indicate that training was tolerated (at a minimum) or that some amount of adaptation has occurred as a result of training; further, a performance improvement after a brief depressed period of heavy training reflects concepts of periodization, founded upon the mechanistic model of Selye’s general adaptation syndrome [21]. Based upon currently accepted training theory, some minor and temporary degree of depression of athletic performance is to be expected at times during any training program ([22], pp. 584–587). At this stage, however, no threshold for concern has been identified by practitioners for classifying a decrease of specific physical performance variables, nor have practitioners determined how much a decrease in one weight room-based performance variable would contribute to an overall performance decrease—for an oversimplified example, no observation has yet demonstrated that a temporary depression of a certain percentage of 1 RM back squat would reliably correspond to a certain change in 100 m sprint times of a sample of collegiate sprinters.

Prior use of the term “nonresponder” has been applied to subjects in studies investigating primarily aerobic training responses, where post-test variables (such as VO_2_max) worsened or remained unchanged following training interventions, or those whose responses did not exceed day-to-day variation [23]. It should be noted that the terms “nonresponder” and “responder” are misnomers when used to refer to training program adaptations. The term response should be used to describe the immediate post-exercise alterations in physiology, etc., and not adaptations to training. However, in adapting this definition to athletic contexts for practical purposes based upon prior use, the term “nonresponder” is defined herein as an athlete who fails to achieve a certain level of performance at the end of a desired training period that was designed to improve specific performance variables. Classification as a nonresponder tends to not be conclusive or permanent—an individual identified as a nonresponder in one variable may demonstrate improvement in other variables [23,24] or adapt well through one training period and not the next. This demonstrates the importance of conducting a regular testing battery and employing a comprehensive monitoring program for athletes. For accurate classification of an athlete as a nonresponder, it is critical to evaluate both the status of the athlete and details of the training program the athlete was exposed to so that an appropriate modification to training or lifestyle may be employed. By conducting a thorough investigation, multiple areas may be identified to explore in order to identify promising avenues to improve training results. Actionable variables may be identified by exploring talent and status of development, fatigue and related influences that affect the training process implemented, habits of the athlete with respect to restorative influences such as sleep, nutrition, and hydration, and how they handle academic and organizational stressors. It is, however, important to consider measurement error involved in obtaining data.

Due to the number of issues referenced above, it is no surprise that objective validation of work quality could be a topic of great consternation for the SC coach. Assessing the effectiveness of coaches is, however, an important aspect of professional evaluation as suggested by Gleason and colleagues [25]. From a management perspective, regularly implementing an evaluation process provides evidence that the organizational investment on infrastructure (in support staff) is paying off, and athlete time is being spent appropriately. To evaluate the SC coach in a fair manner, a thorough analysis of nonresponders should be performed by SC coaches immediately following performance testing sessions and continually as monitoring processes unfold. The purpose of this article is to identify actionable findings, and to suggest possible courses of action in investigating these issues so that results of training and testing sessions can be placed in the proper context, as it influences the professional evaluation of the SC coach. It is important to note that investigation of certain issues may require collaboration with an appropriately skilled support specialist (sport scientist, sport nutritionist, sport psychologist, academic counselor, sport physician, etc.), sport coach, or sport administrator, and may indeed bring about potentially difficult conversations with certain stakeholders or identify organizational processes that need improvement [25]. Without proper investigation of nonresponse to training or concerning maladaptive responses, substantial possibility exists for inappropriate blame to be shifted on the performance staff ([26], pp. 391–392); therefore, we must categorize a nonresponder *based on data*, and minimize the personal bias involved so organizational or personal processes may be corrected.

## 2. Considerations for Testing and Monitoring Programs

Although there is some overlap between testing and monitoring programs, monitoring can be divided into fatigue management and program efficacy. Fatigue management largely deals with the day-to-day alterations in training load and estimates of the athlete’s recovery. Program efficacy deals with performance—physical and physiological adaptation—and whether these adaptations occur at the right times. While the identification of responders or nonresponders is largely dealt with through measures of program efficacy, it should be noted that fatigue and adequate management can play an important role in outcomes. Due to emphasis on the SC context, this discussion will deal primarily with program efficacy and identifying responders or nonresponders.

A thorough testing protocol placed in context of normative data is critical to validate an athlete’s physical development by comparing physical capacity measurements to benchmark values ([27], p. 250). Through use of benchmark values, athletic standards may be employed that allow for contextually-appropriate classification of the athlete’s abilities and used to support goal setting. It is also important to design and implement a comprehensive testing program that includes measures of sport performance, accounts for error, and is performed with sufficient frequency. In addition, a long-term monitoring program may also provide helpful information that may be used to guide training processes.

### 2.1. Measuring Sport Performance

For sports in which results are quantified by values such as time, distance, weight, etc., identifying changes in performance and therefore ‘response’ to training can be relatively straightforward. For example, the performance of a female shot putter improved over the course of a season if her competitive throws increased in distance from 20 m to 21 m. When considering most team sports, a considerable number of variables go into the result (winning or losing) and measuring an individual athlete’s performance change becomes especially difficult. To compare testing measures to sport performance outcomes, coaches may attempt to relate measures to real game statistics (goals, points, yards, tackles, etc.). This offers multiple limitations as game statistics and outcomes can be affected by a host of complex and chaotic variables [28,29]. A more relevant strategy for the SC coach to employ is to identify measures with clear and strong theoretical and empirical evidence for affecting sport performance that are not affected by ever-changing in-game variables (i.e., countermovement jump height). Additionally, the practicality of attaining these measures should be considered, because a measure that is slightly less relevant to a sport may still offer more of an advantage to SC coaches if it can be monitored easily and frequently. This is why the vertical jump is so widely used as a monitoring tool [30]. It is simple to conduct, familiar to athletes, and relevant to many athletic movements [31]. In order for practitioners to determine the most effective measures they should monitor; they must think carefully and critically about how these variables relate to their specific sport and how the tests may be integrated into a cohesive monitoring program (see [20]). Once measurements of interest are selected that are relevant to sport performance and capable of being collected regularly, attention should be placed on identifying what can be considered a true change in these measurements.

### 2.2. Distinguishing a Response from Measurement Error

Measurement error becomes an important concern in identifying nonresponders in sport, as its involvement complicates the determination of whether or not an athlete responded to a training program. A response to a training program may not be identifiable if the true change (signal) is overshadowed by the measurement error (noise). Measurement error can be influenced by the testing device or procedures, in addition to the random within-person variation that is an unavoidable part of human physiology. The amount of error observed is typically specific to the test. For example, a measurement such as body mass can very reliably (<0.5% coefficient of variation (CV)) [32] be measured using a good well calibrated scale; however, small fluctuations in body mass (<1% CV) [33] can occur very quickly and are expected from day to day. Variations in body mass are a common enough occurrence that most coaches are not overly concerned with short-term fluctuations and only pay attention to large magnitude changes or relatively long-term trends. Unfortunately, this process of thinking is not often applied to performance testing. Therefore, we suggest that coaches consider aspects of performance monitoring in a similar manner as they do measures such as body mass. Fluctuations in performance results should be expected, and judgements of response should be determined based on results occurring (1) to a large enough magnitude that it is unlikely to be simply due to error or random variation and/or (2) the changes are trending in a certain direction over time or following a reoccurring pattern. A true non-response from an athlete would then include both a lack of a noticeable positive change above expected variations and an absence of any discernible trend. Small reductions to a performance measure that occur after a single testing session may present insufficient evidence to establish a lack of response, especially if it is within an expected amount of variation. In this way, a similar degree of scrutiny is applied to variables considered performance indicators and those not considered performance indicators.

Measurement error is responsible for an observed result differing from the true value [34]. Reliability refers to the repeatability of a measure; therefore, a measurement that differs considerably from trial to trial (within a session) or between sessions without any logical explanation is considered to be unreliable, and therefore demonstrates substantial measurement error. This error can be due to issues with the measurement device, the practitioner’s skill in conducting the tests, or may be due to biological variation within the athlete themselves. To determine adaptation to a training program, a measurement should display acceptable reliability levels so that true changes in performance can be interpreted. What is considered acceptable reliability will vary, depending on the goals of the measurement; the smaller the error of measurement, the better. 

The reliability of a measurement must also be considered alongside its sensitivity (or responsiveness) [35,36,37]. By definition, a perfectly reliable measure (ICC = 1.0; CV = 0%) does not change from session to session—biological error-free measures are rare in sport settings. If perfectly reliable measures were to be used to evaluate adaptation to a training program, every athlete assessed would be determined a nonresponder over a certain time period. Therefore, performance measures need to be acceptably reliable, measure what they are meant to measure (valid) ([27], pp. 250–252), and respond in sufficient magnitude to be noticeable (sensitivity or responsiveness). True change—and therefore response/adaptation—can be evaluated more effectively with the use of statistics that take into account both the magnitude of effect (effect size) and measurement error (reliability). The specifics and details of conducting statistical analyses for assessing validity, reliability, and responsiveness are outside the scope of this paper; however, practical suggestions and spreadsheets for analysis are available [38,39,40,41,42,43]. The ability of coaches to monitor variables and selected performance measurements, and apply statistical tools to data sets obtained through their own athletes can be valuable because: (1) the controlled conditions of laboratory measurements are not always reproducible outside of the lab, and (2) reliability statistics reported in the literature are parameter or point estimates [44], meaning the true value within a population can vary from the value reported in the paper (ideally represented by confidence intervals). 

### 2.3. Improving the Reliability of Measurements: Balancing Quantity and Quality

Measurements are reliable to the extent that the same value is derived with each measurement. Precision reflects the variability of the measure around its mean, not whether the mean is the true mean. Thus, measurements should be as valid, reliable and precise as possible. Pre–post testing strategies only assess an athlete’s performance at two-time points, either of which could be influenced by a multitude of variables. Consider a collegiate athlete whose second testing session ends up being scheduled on the same day as a major exam. This athlete could have potentially been developing positively throughout the entire training program; however, the influence of an outside stressor (exam) may prevent them from effectively revealing those performance improvements at that specific time point [45]. Therefore, the use of multiple mid-test sessions may be valuable ([27], p. 250). Frequent and consistent collection of monitoring data can help discern true change from random variation by allowing for recent results to be compared to historical data, which could help identify expected (normal) amounts of variation. Additionally, an increased frequency of data collection prevents the need for determining a response based upon pre–post changes and may reduce error [46]. On the other hand, coaches must strike a practical balance in testing frequency—too frequent testing can increase cost (for some variables) and introduce more variation if the athlete ceases making a maximum effort because testing has become monotonous. Determining optimal test frequency is likely dependent on the level of athlete tested, the quality of data being collected, and the organization’s resources. Practitioners should seek a practically manageable process that allows for sufficient identification of trends or patterns in the results that may be useful for informing future training decisions [47] without undue stress upon the athletes. 

While the frequent collection of performance data may be beneficial in many circumstances, a high quantity of low-quality data will only add to the noise and make it more challenging to be used to identify true response. The quality of data is generally determined based on its fit for the intended use and should be accurate, consistent, complete, and up to date [48]. Rigorous standards should be set for testing scenarios to collect as high-quality data as possible and minimize measurement error. Testing considerations such as the time of day [49], instructions given [50], and warm-up used [51], among other factors, have all been shown to affect performance results and therefore need to be controlled for. Additionally, steps should be taken to create procedures for entering, storing, and using the data so that minor faults in data management, such as having duplicate entries or entering values with differing units of measurement (kilograms vs. pounds), do not end up impeding its potential usefulness for determining and evaluating athlete response.

### 2.4. Decision Making

It is also important to acknowledge the reality that some amount of subjectivity is often applied in evaluating sport performance particularly in team sports (e.g., soccer) or individual sports with panel evaluators (e.g., gymnastics) where assessment of individual performances may be heavily influenced by personal bias of sport coaches or judges. If an SC coach is working with a sport coach who feels that an athlete is not developing adequately, the SC coach must rely upon data to form their own opinion. In truth, if an athlete is progressing well in areas of physical development, but progressing poorly in application of sport tactics, there is little the SC coach can do within their own domain to help the athlete [52]. Certainly, a data-centric discussion with the sport coach would be required in this situation in order to evaluate the player’s physical status without bias. A variety of information sources have been recommended for SC coaches to consider in designing and influencing the training process [25,52,53]. Recent opinions have highlighted that physiologically-derived concepts are often the primary emphasis of SC decision-making processes (and educational programs), highlighting the necessity of broader, more holistic decision-making processes due to the interdisciplinary nature of coaching [53].

**Figure 1:**
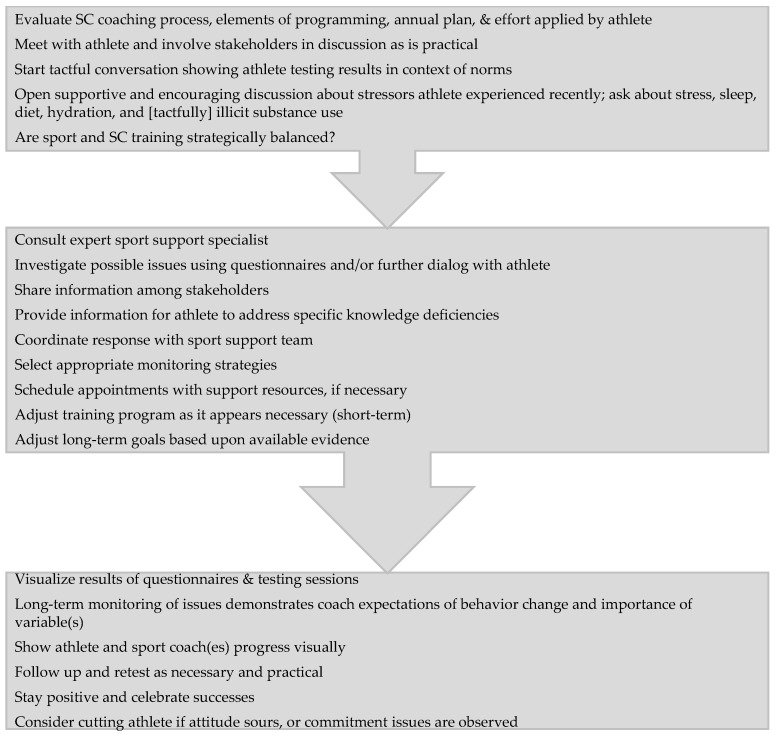
Flowchart of investigation and intervention.

It is important to consider a range of possible threats to the quality of information obtained in the testing data, and to keep the developmental nature of the athletes involved in context. If a thorough investigation is conducted, various explanations of an athlete’s lack of progression may be found. This process requires a formal or an informal meeting between the SC coach, the athlete, and the sport coach (Figure 1). An initial discussion of the below suggested areas of influence and concern may lead to a basic understanding of the athlete’s stressors, habits, and level of commitment. Further investigation may also be possible through the use of surveys and referrals to sport support specialists that investigate the areas of influence and concern listed below. 

## 3. Developing Strategies to Help the Athlete

From a SC perspective, when an athlete is found to be a nonresponder, first the SC coach should look to the structure of the training program and evaluate programing structure and service delivery using self-reflection practices—these are mechanisms used to question processes involved in coaching (what, how, and why?) and apply critical thinking to foster improvement in service delivery [53,54]. Provided that the program supports objectives that are clearly defined and targeted based upon available evidence, additional explanations for nonresponse should be sought. Subjectively, many of the issues (sleep, the dog ate the homework, family problems) identified in this review may be recognized by engaging the athlete in conversation or from use of a variety of questionnaire tools on an acute or recurring basis [55]. While several resources have been validated, many coaches have developed custom forms and tailored them to the sport context and information sought ([56], pp. 90–100). Coaches are referred to Saw’s work [57,58,59] for detailed recommendations on monitoring surveys, including designing custom tools; keys to success with these strategies that may be applicable to the SC context involve using brief questionnaires that target the desired information and use language the athletes are comfortable with. Because of the reality that some athletes will avoid revealing problems, coaches should be wary that this is a possibility and therefore repeated delivery of a survey is necessary. The reality of working with athletes suggests that many do not pursue help-seeking behaviors, despite struggling with multiple competing responsibilities [60]. In addition, social stressors encountered in adolescence and young adulthood may prove challenging to cope with for many athletes, particularly in light of additional stressors that come about from sporting performance [55]. Because of these issues, recommendations for consistent monitoring of athletes have been made [55]. 

Coaches may align support resources to assist with psychosocial, academic, or professional pursuits [41,61,62,63]. Because of regular access and proximity, the SC coach may be in a strong position to identify athletes needing support so that appropriate resource may be applied (e.g., referral to academic counselors so that tutoring may be arranged) [45]. Recent research in sport science has identified the value of identifying the existence and sources of stressors so that appropriate steps may be taken to help the athlete focus, recover, and adapt [55,63]. Specific training strategies may be more appropriate for an athlete that does not adapt (respond). Providing that the lack of response cannot be traced to outside stressors, training programs can be individualized, particularly with assistance of a sport scientist [64]. 

## 4. Areas of Influence and Concern

### 4.1. Talent

Talent is a concept widely applied, but often poorly defined. Breitbach and colleagues [65] recently translated Hohmann and Seidel’s definition of a talented athlete as a person whose performance capabilities are above average—compared to an appropriate reference group—that has achieved or is projected to achieve a certain performance level. Talent is largely genetically determined, but subject to development influences also, making the topic complex; however, genetic components typically overrule nurture-related components in explaining the development of high-level athletes [66]. While biological factors constitute a major component of why an athlete can perform at a high level, the association between genetic endowment and performance is unclear. In a recent meta-analysis, Zempo and colleagues [67] observed some variability in muscle strength-related phenotypes, and this was reported to vary between tests used. While the topic has yet to be elucidated well, the genetic potential for adaptation presents fertile ground for athletic ability research. At this stage, it appears that opposed to the idea of genes in isolation, polymorphisms and gene networks may be responsible for the variability of physiological adaptation in both strength-related and aerobic adaptations [5]. Unfortunately, genetic testing is not yet a viable approach for talent identification [24]. Although, nature apparently plays a predominant role [66], strategies for obtaining and analyzing genetic information have not provided reliable information that completely explains why a good athlete rises to the top, compared to traditional methods of talent identification [68]. Further improvement to the effectiveness of talent identification processes may require association between physical variables with cognitive-perceptual and psychological variables necessary to perform well in the sport, better identification of “*what does talent look like?*” as well as the realization that talent is likely to emerge over time in the athlete, instead of being perceived as a fixed quality [69].

An important point to note, however, is that rare severe maladaptive tendencies can be identified using genetic testing. Certain genetic variations, including sickle cell trait and other more uncommon variations, have been implicated in multiple events of exertional rhabdomyolysis, indicating acute and potential future maladaptation to training [70]. Accordingly, any individual event or cluster of rhabdomyolysis should be of major concern to SC coaches, sport medicine staff, and administrators, and as such they are recommended to pursue genetic testing to rule out further issues for the athlete(s).

*Actionable points:* Coaches are advised to exercise patience with athletes as they develop, due to the reality that existing methods of talent identification—particularly early identification—have substantial error [69]. Sport medicine professionals should pursue genetic evaluation of athletes who experience exertional rhabdomyolysis to rule out the possibility of recurring susceptibility to injury. Further actionable genetic testing options may be difficult to support based upon present minimal understanding of the sporting implications of the human genome. 

#### Impact of Somatotype

Anthropometry measurements are common in the field of SC ([27], pp. 262–263), however the physical dimensions of athletes may be evaluated using more detailed methods. Somatotype is a 3-number index for classifying physique into categories of endomorphy (broad frame, higher body fat), mesomorphy (medium frame, more muscular), and ectomorphy (slender, less muscle mass) [71]. Somatotype is strongly related to heredity, particularly mesomorphy and ecotomorphy [72], and can have considerable influence on performance. Athletes of specific builds have been associated with greater success in certain types of sports, however physical requirements among positions in team sports may vary [73,74]. Somatotype has been found to be related to physical performance variables, including aerobic capacity [75] and anaerobic performance [76]. Physical build has been found to influence the amount of muscle mass may develop in male subjects, with slender individuals gaining less fat free mass [77]. It is therefore likely that empirical evidence of physical adaptations may be used to effectively indicate talent in the acute setting, however long-term evaluation programs demonstrating trajectory of development among talented athletes are uncommon among sporting organizations.

*Actionable points:* A robust testing program may serve to aid coaches in decision-making processes, particularly those that influence roster strength. Somatotype is recommended to be included in athlete profiling processes. 

### 4.2. Physical Development

Particularly common at the collegiate level in the United States (but not exclusive to it), the SC coach may inherit athletes in one recruiting class with substantial strength deficiencies and technical deficits, while others may be better-developed. Poorly developed athletes present challenges to the SC coach that require further resources—particularly time and patience. Because of challenges to an athlete’s time management skills, an additional time commitment can present further stressors to an athlete at the college level [45]; this highlights the importance of working with a qualified SC coach during high school years. Decisions of sport coaches and administrators play a large role in provision of resources to prepare athletes for the next level; therefore, if insufficient resources exist in a youth or high school setting, appropriate hiring measures may be made by the organization’s leaders in order to provide adequate services for athletes [25]. Issues such as poor physical development may indeed increase the odds of a U.S. college sport coach’s decision to “redshirt” a player (sit out a year without using one of 4 available years of athletic eligibility). This potentially results in a greater cost to a U.S. university sport program; however, it may be in the athlete’s best interest in many cases.

When an athlete presents with lesser physical development, this can often be demonstrated via visual means in context of sport-relevant and developmentally-relevant norms. Performing a battery of physical tests, including advanced anthropometry techniques, will allow for an objective assessment of the athlete. Several resources are available providing guidance on demonstrating profiling methods visually, as well as practical, low-cost analysis methods that can be performed using Microsoft Excel [41,78,79].

Athlete selection literature suggests that sport coaches’ ratings of athlete talent are inconsistent between coaches and indicates a need to seek more objective selection procedures [80]. It is therefore to the SC coach’s advantage to highlight physical ability/capacity variables, providing a collection of empirical indicators that can demonstrate that the athlete is progressing favorably. Because many qualities of physical performance (maximum strength, 10 m running speed, etc.) are often within the reach of the SC coach, these are variables that are advisable to emphasize [52]. 

*Actionable points:* Qualified SC coaches should be employed at the high school level to support athlete development. In the event that an SC coach is pressured by a sport coach regarding an athlete’s progress in development, highlighting the magnitude of a player deficit compared to normative data may help validate a need for patience and also help to prioritize training.

### 4.3. Balance of Stressors and Impact of Fatigue

As we consider the underlying mechanisms upon which modern methods of sport training are based, we can conceptualize stress applied to the athlete from which physiological and psychological outcomes can result. The application and accumulation of training and life stressors may indeed result in performance fluctuations and can temporarily depress performance following times of heavy training stress due to fatigue [21]. 

Minor short-term depression of performance as a result of highly concentrated training is seen as a functional component of managing training workloads [81]. Variation in performance may be observed in available reviews reporting performances of high-level athletes [82,83]. As athletes develop over time, adjustments to training programs are necessary, including application of progressively greater training stress than the athlete has experienced beforehand ([84], p. 228), which is best guided by a monitoring program [85]. Keeping the athlete’s training history in mind may shape coaching decisions of how hard to push the athlete at a given time and with which strategies. A novice athlete may obtain good results with a more basic program that focuses on skill development, while a more advanced athlete may benefit from more advanced methods and aggressive strategies.

Various issues may result from poor training design. A general model commonly discussed among sport medicine professionals is the concept of an appropriate training dose, where underloading, overloading, and insufficient recovery may increase injury risk to tissues or increased risk of illness [86,87,88]. In reality, this “optimal” dose is likely individualized to some extent. However, it should be noted that team-level injury trends have been associated with training and competition workload [89].

*Actionable points:* SC coaches are advised to consider the broader range of stressors an athlete is exposed to and the training history the athlete. Does evidence suggest the loading is too high or too low, too frequent, or too complex? A monitoring program may be of particularly helpful in identification of issues (discussed later). 

#### Optimizing Workloads

An optimized loading strategy should result in a desirable long-term trend toward improvement of a particular athletic quality, while chronic underloading or excessive loading may not result in desired improvements [21]. Positive recovery-adaptation also requires the appropriate balance of recovery strategies with training; it is generally accepted that maladaptation occurs as a result of imbalance in some facet of the training puzzle, which may include a rate of increased loading that is too rapid for the athlete, underloading, or overloading [86,87,89]. 

Training strategies employing autoregulation tactics have become popular in the SC field. Autoregulation is a mechanism where training loads are adjusted based upon information gained during or prior to the training session in an attempt to gauge the effects of fatigue upon the athlete at present and modify the training session according to what the athlete is likely to be able to accomplish and recover from [90]. Examples include using countermovement jumps, fatigue surveys, or intra-workout barbell velocity to adjust loading intensities or volume of the present session or those within the microcycle [88]. The reliability and validity of certain technological tools [91,92,93] that may be used to inform autoregulation processes has been investigated, as well as subjective methods [59,94]. While optimal strategies for use in a particular setting remains a debate, it is important to note that anecdotally all SC coaches use some form of autoregulation as part of typical variation strategies; therefore, the practice has been largely universal based upon necessity of adapting training programs to predictable and unpredictable demands placed upon athletes. SC coaches are strongly encouraged to investigate the limitations of methods they choose to employ so as to minimize error from the device or subjective strategy. 

An additionally relevant consideration for the workload optimization is the observed outcome compared to intended purpose of a training stimulus for an athlete. For example, if a training phase is specifically designed to induce a maximum strength adaptation and fails to bring that about, the coach should consider investigating the possibilities as to why a lack of expected adaptation occurred. 

*Actionable points:* Evaluate programing based on monitoring and testing data in context of error. Cautiously apply autoregulation strategies, keeping the training plan in place. 

### 4.4. Academic Stress

While training-related stressors comprise a major component of overall stress, athletes do not train and exist in a vacuum. A broad range of sources and intensities of stressors exist, and these may be biological, psychological, and social in nature [45,95]. In addition, many stressors are related to academic pursuits [45,96,97]; educationally-derived stressors are unavoidable is sport systems featuring integration of sport with scholastic and university institutions. Some of these stressors, however, may be predictable based upon time of the academic term and the rigor of academic commitments. Certainly, clues leading toward effective management of training stressors may be used strategically by coaches in planning training, as surges (e.g., term papers, mid-terms, finals) in academic responsibilities may be foreseeable within the near future. Practical strategies involve surveying athletes to investigate their level of academic concern and time management [45]. For example, conducting a survey within an athletic team in which athletes rate their immediate or pending (within a week) perceived academic stress on a scale of 0 (no issues at all) to 10 (experiencing great anxiety due to volume or challenge of assignments) is a low-cost option that may help identify issues relevant for intervention. 

Coaches must consider that the athlete may be particularly sleep-deprived during stressful academic periods [96]; actionable points beyond reducing practice time and training requirements (duration and intensity) may be difficult to identify in athletes who are committed or struggling students. Evidence exists of injury risk increasing during times of exams [96,97], which suggests coaches should consider reducing training loads during these periods. Specific guidance to effectively offset academic stressors by way of programming adjustments has yet to be developed and is likely individual in nature due to the level of academic stress the athlete experiences.

*Actionable points:* Practice time or training time may require reduction during times of academic stress. Athletes should be mentored to improve time management skills. Athletes may be referred to academic counselors for further assistance.

### 4.5. Sleep

Sleep has received substantial attention in sport science in recent years as a primary restorative resource [98,99,100,101,102]. Several reviews have detailed the prevalence and causes of sleep deficits among athletic populations, as well as providing strategies to improve sleep [98,100,103,104,105,106,107]. Overall, sleep extension (sleeping longer), napping, and improving sleep hygiene (behaviors that improve sleep quality and quantity) may be viable objectives for athletes at many levels. The variables commonly observed in sleep diaries and questionnaires are: time to bed, time awake, total sleep time, sleep latency (time required to achieve sleep), number of interruptions, details of napping, subjective sleep quality, and daytime sleepiness [106]. The reader is referred to [106] for specific recommendations on tools and processes of sleep monitoring and [100,108] for suggestions on how nutritional interventions may improve sleep.

Numerous benefits can occur during sleep, including enhanced hormonal signaling associated with physical growth and restoration, psychological restoration, memory consolidation, immune system maintenance or enhancement, along with a number of other beneficial phenomena [107]. Athletes observed acquiring insufficient sleep have demonstrated reduced performance, possibly due to impairment of nervous system function [102]. Though long-term direct association with injury is debated, acute trends of insufficient sleep have been observed preceding incidences of injury [109], theoretically due to reduced awareness or increased reaction times in stressful situations. Pertinent to athletes, competition schedule has been found to affect sleep, with substantially reduced sleep (vs. training) demonstrated by athletes competing in night games [110]. In addition, travel has been found to reduce sleep quality and quantity, particularly across multiple time zones [107,111]. This has been reported as an issue of concern by elite athletes [112]. Time zone changes are often a part of high-level athletic pursuits, therefore athletes likely to be subject to time zone changes are advised to develop tailored strategies to cope with this issue [108,112].

Though a range of 7–9 h of sleep is frequently recommended, individual variation and training phase variation is likely [103]. Fox and colleagues [104] recommended a regular minimum threshold of 8 h sleep per night as a meaningful target for injury risk mitigation in young (14–25 years old) athletes. Some younger athletes in periods of heavy training may require up to 12 h per night [113]. General guidance suggests if the athlete is unable to remain wakeful in necessary meetings, classes, etc., then a sleep deficit may be evident. 

Several studies have demonstrated improved sleep hygiene following an educational intervention [114,115,116], suggesting that sleep education may be a necessary topic for athletes at all levels. For practical application, sleep improvement strategies suggested by Halson [105] and Bird [103] are shown in Table 1. Because of limited budget and time to pursue devices such as polysomnography or actigraphy, sleep monitoring surveys or sleep diaries may be most appropriate for application by the SC coach and athlete seeking to explore sleep habits. The reader is recommended [105] to investigate Ibanez’s [117] evaluation of sleep surveys and diaries for further detail. 

It is important to note that high rates of obstructive sleep apnea (OSA) have been reported in athletic populations [118,119]. Because of the likelihood for sleep impairment as a result of OSA, large athletes with large neck circumferences may particularly benefit from basic screening practices and follow-up treatment [120].

*Actionable points:* Sleep education may improve athletes’ practices in the short-term; follow-up analysis and education is likely necessary. Sleep diaries or surveys may help identify issues. The SC coach is a potential educational resource for athletes on basic sleep education due to proximity and rapport, and may be an appropriate person to immediately identify athletes in need of intervention. Sleep apnea is common in athletic populations, particularly among large athletes, and may require medical intervention.

#### Screen Time, Blue Light, and Further Challenges

Studies examining the influences of electronic devices on sport performance have been found to be inconclusive at this stage [104]. Some evidence in general population (young adults 20–24 years old) has found associations between medium and high rates of computer and mobile phone use and sleep disturbances, particularly for males, while symptoms of depression were associated with electronic devices for extended periods without breaks for females [121]. Until more is known, athletes may investigate their own responses to screen time using a sleep diary. For example, exploring the association of evening duration of screen time and screen-off time with time to sleep, sleep latency, and sleep quality may be useful.

Exposure to blue light from screens and artificial light may have effects on the sleep-wake cycle and affect sleep quality [122]. As a result, the use of amber lens glasses to block blue light in the evening has been investigated. Recreational athletes wearing amber glasses in the evening have shown improvements in sleep—by subjective ratings, but not actigraphy—when paired with morning light exposure protocols [123,124]. Due to the reality that many athletes are students, restricting screen time may be an impractical option—particularly due to the technological shift in education brought about by the COVID-19 pandemic. It is likely that internet-based meetings and educational offerings are here to stay. Further research is necessary to confirm the effects of light manipulation strategies; at this stage SC coaches should be cautious recommending minimally-supported strategies.

Another challenge is the behavioral aspects of changing sleep. Halson [106] identified applications of behavior change theory to sleep habits, highlighting that long-term monitoring or follow-up investigations may be important to confirm habits have indeed changed. In addition, medical or psychological interventions may be necessary for athletes with sleep disorders, therefore SC coaches are encouraged to coordinate efforts with sport medicine practitioners if more aggressive solutions appear necessary [106]. One sleep workshop model designed for and implemented with U.S. collegiate athletes by Kaier and colleagues [125] showed some promise.

*Actionable points:* Follow up investigations may be necessary to investigate sleep in athletes. Coaches should be sensitive to the impact of early practices, late meetings, and academic work upon sleep.

### 4.6. Nutrition

Nutrition strategies may differ for an athlete based upon the training schedule and the time of year [63,126]. In addition, body composition goals may influence food intake for athletes in the offseason, leading to small or major changes in calorie intake ([127], pp. 216–218). Unfortunately, many athletes are often nutritionally unprepared, in that their knowledge is not sufficient to construct an appropriate diet on their own, demonstrated by poor performance on nutrition surveys [128,129]; however, educational interventions have shown some promise in improving athlete nutrition knowledge [130]. 

Specific maladaptation is possible as a result of heavy training paired with a poor diet. Representing a worst-case scenario, one case study detailed the case of a 16-year-old national level swimmer diagnosed with exertional rhabdomyolysis after training heavily while consuming a poorly controlled lacto-vegetarian diet with a negligible protein intake [131]. This case study highlights the importance of nutrition education in athletic populations.

Basic guidance has been longstanding in S–C education resources; for instance, the NSCA Essentials textbook provides a variety of methods for estimating caloric needs in athletic populations based upon activity level and guidance for optimizing body composition ([127], pp. 126–127). In under-resourced sport programs that lack access to a sport nutritionist, many SC coaches may find themselves in a position of serving as a nutrition resource for athletes; this may be appropriate to a certain extent, as SC coaches have been found to possess the greatest nutrition knowledge of non-nutritionist athletic support staff at the college level [128]. However, it is important for coaches to understand the threshold of serving as an expert (meal planning) and a consultant providing general guidance on food and supplements. Because of specialty training, it is only the licensed Registered Dietitian that is trained to provide comprehensive diet planning services [132]. This is a skill developed by academic training and a formal supervised internship, and practice in this field is usually governed by state laws. In addition, the sport nutritionist undergoes another specialty qualification in the U.S., such as the Certified Specialist in Sport Dietetics (CSSD), for example, allowing the specialist to focus on athlete-specific nutritional needs [132]. SC coaches are advised to involve an appropriately-credentialed sport nutritionist if detailed meal planning education is necessary. 

If a SC coach suspects an eating disorder, they are advised to alert sport medical personnel so the athlete may be connected with appropriate medical experts to explore the issue. It is also recommended for athletic programs to employ an experienced and appropriately-credentialed sport nutritionist, at least in consultant role. SC coaches serving as the nutrition resource for entire athletic department can be a cumbersome duty outside their primary skills. This is one organizational area that may be identified during a formal evaluation [25].

Because of the nature of sport competition, athletes experience a variety of nutritional challenges. Travel impacts food availability, and for many athletes foreign cuisine can present issues that may affect performance [112]. Coaches are encouraged to maintain open communications with athletes about food quantity, quality and access during travel so that effective strategies may be devised. The services of a sport nutritionist may be of substantial value for teams that travel over long distances. 

*Actionable points:* Nutrition education of coaches and athletes is necessary. Adapting the training load to nutritional needs is warranted. Travel may introduce nutritional challenges; therefore, coaches should work with athletes to ensure sufficient resources are available. Consulting a sport nutritionist is required to optimize diet.

### 4.7. Hydration

Evidence suggests endurance performance, repeat sprint performance, strength and skill-related performance may be negatively impacted by a hypohydration threshold indicated by loss of 2–3% body mass during field and laboratory testing sessions [133,134,135,136]. Individual effects may vary, and they are related to athlete sweat rate, sweat electrolyte concentration and possibly differences between the two [137] or some other unexplored physiological phenomena. Drinking sufficient water to recover body mass has been proposed to be an important aspect of recovery, as is consuming electrolytes, so that the intracellular environment is normalized ([138], pp. 72–77). It is important to note that hyponatremia has been observed in large proportions (10–13%) of long-duration endurance (ironman) athletes following competition [139] and implicated in deaths of numerous athletes [140]; this is an issue because a range of concerning neurological symptoms (confusion, brain swelling, seizures, and death) may result from severe hyponatremia, and rapid correction of severe hyponatremia can result in damage to brain myelin tissue [141]. It should be noted that hyponatremia can be caused by too much electrolyte (e.g., Na+) loss or by too much water intake, even during exercise [142]. 

While many detailed profiling options such as sweat sodium and water loss investigations may be outside the scope of a SC coach’s typical duties and skills, SC coaches often share duties with sport medicine staff in delivery of basic education; assisting with investigation of potential issues is certainly reasonable to expect from a SC coach as time allows. Seeking services of a sport scientist to investigate team-level hydration issues is necessary if the SC coach lacks the time and expertise to attend to this issue. 

*Actionable points:* Hydration education is required. SC coaches should work with sport medicine and sport coaches to ensure athletes are conducting weight monitoring.

### 4.8. Alcohol

Similar to the general population, alcohol use is common in athletic populations [143]. Preliminary evidence indicates that ingestion of alcohol may impair recovery for many athletes, though more robust ecologically valid evidence is still lacking. One study reported that ingestion of 1.09 mL/kg per fat free mass of vodka during the 10–20 min following a resistance training session was sufficient to blunt hypertrophic cell signaling within 5 h of training in male resistance-trained young adults, but not females [144]. Therefore, in the short-term, alcohol consumption may impair mechanisms that induce protein synthesis in the short term for males, however further research is necessary to understand the implications of alcohol upon recovery and adaptation over a longer term; exploring potential differences between female and male athletes is also necessary. 

Tangible effects of social alcohol use on long-term development of athletic skills are not known at this stage. Barnes [143] suggested that mild alcohol use (up to 0.5 g/kg body weight) is not sufficient to impair recovery. Alcohol is known to induce dose-dependent toxic effects upon nerve tissue, and long-term heavy alcohol use is associated with numerous health conditions, including many that degrade movement competency [145]. In addition, alcohol use has been observed to temporarily suppress immune function and suppress formation of anabolic hormones in male athletes [143]. Importantly, heavy drinking may induce short-term dehydration, which could affect next-day performance and health [143]. 

The effects of alcohol consumption upon sleep have been well-established in healthy adult populations, with generalized results including sleep architecture changes such as sleep onset promotion (favorable), reduced rapid eye movement sleep (unfavorable) and circadian rhythm interaction (likely unfavorable) [146]. These were generally replicated in one study including healthy young adult (18–21 year-old) subjects, suggesting only minor differences between these subjects and other adult populations [147]. Heavy alcohol use is likely to negatively affect sleep, leading to reduced recovery [143]; in many cases, this may be a result of worsened snoring and obstructive sleep apnea [148].

*Actionable points:* Athletes need alcohol education. Providing alternative activities for stress relief is recommended, particularly those that foster strong sleep routines.

### 4.9. Other Recreational Substances

While it may be an uncomfortable point for athletes and all stakeholders, discussion of the negative effects of recreational drugs is necessary for holistically developing athletes. Because of the stressors athletes experience in addition to their non-athletic peers, athletes may use illicit drugs to relieve stress, anxiety, and manage pain; use is widespread in some sport subcultures [149]. Though evidence suggests cannabis (marijuana) use, for example, results in acute impairment of memory, coordination, and judgement [149], insufficient ecologically valid evidence exists to demonstrate long-term performance impairment or enhancement [150]. Similarly, despite some preliminary supportive evidence in animal models, insufficient evidence presently exists for use of cannabidiol (CBD), a non-intoxicating chemical compound derived from cannabis, for easing anxiety, reducing inflammation, management of concussions, or other temporary interventions that may aid athletes [151]. In their review, Schierenbeck and colleagues [152] overviewed the effects of cocaine, MDMA (ecstasy), and cannabis on sleep, indicating that major illicit drugs all affect sleep negatively in some way. Effects range from acute sleep reduction and disturbance (cocaine, MDMA), and effects may last as long as 48 h after administration (MDMA). Acute cannabis use appears to increase sleep onset and increase sleep architecture variability, with unknown results long-term. 

Considering the nature of social use of illicit substances, impairing the restorative effects of sleep during weekends may rob the athlete of their best opportunity in the week to catch up on sleep if their schedule prevents regularly obtaining sufficient sleep or an acute deficit is acquired. Because of the developing nature of athletes, coaches should help athletes develop strong coping skills so that chronic behaviors that favor long-term health may be fostered [55].

*Actionable points:* Coaches are advised to steer athletes away from using alcohol and illicit drugs for stress management in favor of more appropriate substitutes that favor recovery and productive stress relief. Considerable privacy issues exist regarding the illegal status of illicit drugs; therefore, coaches should be cautious in their approach in obtaining information from athletes regarding this topic in the interest of athlete privacy and stay abreast of changing laws regarding cannabis use as they pertain to team rules. 

### 4.10. Organizational and Miscellaneous Stressors

The social fabric of team sports is indeed a complex area to consider. Social conflicts with teammates or coaches and results of aggressive coaching strategies may affect an athlete negatively [55]. The reader is referred to [112] for discussion of organizational stress perceived by a sample of 10 male international-level athletes. Areas of stressors included performance, environmental, personal, leadership, and team issues (coach-athlete interaction, playing position within the team, personality conflicts, etc.). Even changes in competition timing may affect injury status as athletes prepare [112]. Additionally, negative interpersonal relationships may also induce stress to the athlete and affect performance [153], so the psychological aspects of the athlete are an area worth maintaining ongoing awareness. Ultimately, recommendations for successful sport organizational culture include proactively pursuing information to identify issues and to obtain athlete perspectives, and supporting athletes with educational/counseling interventions to help them respond effectively to stressors [55]. 

Multiple tools to evaluate athlete stress levels have been designed, which may be used to help SC coaches identify issues outside of their domain. In their review, Nässi and colleagues [154] detailed available psychological survey tools and provided insight on their use for application in monitoring training and recovery. Some tools in this area have > 20 questions and may require several minutes to complete, therefore this may necessitate less frequent surveys due to time constraints. However, more recently several short surveys (APSQ, DALDA, SRSS etc.) have been developed (<10 questions) with good validity and reliability that may be given more frequently [155], and it is possible that these short forms could be administered more frequently. Coaches are advised to keep results as confidential as possible so as to foster trust with athletes and increase the likelihood of athlete honesty in responses. Assistance from a qualified sport scientist may be necessary to interpret some survey results and design appropriate interventions.

Fostering and maintaining a supportive climate has been recommended for the SC coach [156], and noted as an important component of behavior of successful SC coaches [157]. Providing mentorship to help athletes holistically grow and develop successful habits has also been attributed to the successful SC coach [158]. Further, some psychologically-oriented skills such as the provision of emotional support have been reported to be valuable for SC coaches; this constitutes an important component of job tasks due to ability to develop rapport with the athlete [159]. Basic sport psychology-related training may also be provided by the SC coach, in absence of a psychologist [159]; this task set extends to include the use of context-specific mental imagery [160]. SC coaches have been recommended to serve as a resource to relay information to other support services within the organization [159], therefore when a SC coach has reached their professional limitations in helping an athlete or lacks the time to assist, they should seek to connect the athlete with a specialist resource. 

Coaching methods defined as maltreatment may be observed periodically in sport settings [161], and SC coaches may find themselves as witnesses in events or develop awareness of problems from discussions with or proximity to athletes. SC coaches are advised to be cautious in handling these events for political reasons, however a formal evaluation session with a supervisor would be an excellent time to bring up acute or chronically observed issues [25], especially within a college or high school athletic department where administrator oversight of sport coaches may be challenging. 

Consistent with the authors’ experiences in U.S. high school and college settings, athlete financial stress may occur that increases psychological distress and can be manifested as food shortages. Coaches should not forget financial issues are always a potential issue for athletes in youth sport and in the U.S. collegiate system (which presently restricts athlete earnings), therefore the realities of financial stress could present substantial distraction from performance—and regularly affect sleep. 

*Actionable points:* Coaches are advised to remain positive with athletes who are struggling to improve performance. A variety of stressors may be perceived by the athlete and be apparent once investigation begins; therefore, coaches should be open to criticism if it is warranted and processes should be amended if necessary.

### 4.11. Motivation

Skinner and Stewart ([162], p. 117) define motivation as being “sufficiently energized to engage in some form of activity”, and further characterized it as “a desire or want that energizes and further directs goal-oriented behavior.” Anecdotally, motivation is associated in a linear fashion with performance and certainly a noticeable quality present in all high-level athletes with whom we have worked. Motivation is a complex construct, in that it is situational, goal-directed, and greatly variable within and between people ([162], p. 117). 

In spite of its complexity, motivation may be considered one of the most important variables in sport that impacts not only the performance of an athlete but also their sport experience [163]. Particularly important to enhance sport performance is the development of strength and improvement of rate of force development through regular training [164]. With the role these critical qualities play in sport, it is important to consider how motivation can impact an athlete’s willingness to train, which ultimately affects the quality of the stimulus applied that is intended to improve sport performance. Value may be found in coaches specifically observing athletes’ tenacity and intensity with which they pursue training tasks, in addition to investigating athlete thoughts about their performance and goals [165]. 

#### 4.11.1. Drawing Insights from Motivational Theory

Motivational theory research explores why people are or are not motivated. The most cited theories include self-determination theory and achievement goal theory [166]. These research lines lay the foundation for understanding what motivates athletes. 

Self-Determination Theory’s (SDT) basic concepts revolve around actions being intrinsically and/or extrinsically motivated [163,167]. Intrinsic motivation is considered internal to the athlete and unaffected by reward or punishment ([168], pp. 161–162), because of the satisfaction and pleasure it provides [167]. This can be extended to intrinsic regulated motivation, which involves participation in sport because the activity is satisfying and interesting [169]. Extrinsic motivation, on the other hand, is sourced from something external to the athlete such as a tangible reward such as social approval or avoiding punishment ([168], pp. 161–162), both of which constitute external regulation. In addition, an athlete may pursue sport in order to seek avoidance of negative feelings (introjected regulation), or to attain personally valued goals (integrated regulation) in order to obtain recognition or approval [163,167,170]. In their discussion of motivation, Deci and Ryan [171] also include amotivation, which is defined as a lack of intent to engage in a particular activity [169].

Relative to SC coaches, SDT proposes that persistent behavior and psychological well-being are impacted by the quality of motivation toward a particular activity [169]. This may have long-term consequences in effort applied in sport training contexts, especially if reduced-quality training stimuli are consistently applied by poorly motivated athletes. In addition, Sarrazin and colleagues [170] noted that athletes who dropped out of sport activity were much less intrinsically motivated, and more externally motivated or amotivated when compared to those who remained in the sport. When placed in context of an athlete’s career, intrinsically motivated athletes may indeed be what coaches are hoping to foster or select. 

Achievement Goal Theory (AGT) revolves around the personal motivational elements (achievement goals) and sporting contextual elements (goal structures), and holds that these goal structures (in the form of environmental cues) may influence the achievement goals that athletes select [172]. The achievement goals govern the achievement beliefs, which in turn guide athlete decision-making process and behaviors [173]. As a result, athletes are advised to set goals that are specific and difficult, but attainable. Goals should be monitored by coaches; this can be an influential practice for motivating athletes, particularly with coach feedback over time [174].

#### 4.11.2. Determinants of Motivation

In accordance with SDT, there are three basic needs that underpin motivation which must be considered: competence, autonomy, and relatedness [167]. Competence can be determined by how well an athlete can perform a skill, and is formed by their experiences within the specific social environment [167]. Autonomy refers to self-regulation and is reflected by behaviors that are performed intentionally and by choice; relatedness refers to the connection an athlete feels as a result of being included and cared for by others in the context [167]. When conditions are perceived to satisfy the above three needs, motivational levels towards the activity will be higher; without them, motivation may be reduced [163]. 

Rooted within the above basic needs, situational and contextual determinants of motivation exist. Situational motivation is described as the motivation an athlete experiences while engaging in a specific activity [175]. Situational determinants of motivation include rewards and awards, competition, negative and positive feedback, success or failure, and choices, while contextual motivation refers to motivation toward a specific situation [175]. A contextual determinant of motivation includes the coach and teammates, the motivational climate, scholarships, and sport structures [175]. Certainly, both situational and contextual motivation could be reduced by organizational stress or interpersonal issues among a team, for example. 

Some connection has been made between motivation and athletes’ birth order, suggesting a possible social influence. In one study, 65% of national-level weightlifters (*n* = 20) were firstborn, tending to be more intrinsically motivated than later born athletes [176]. Another study of Australian and Canadian athletes (*n* = 304) competing in 33 different sports found only 10% of elite athletes were firstborn [177], indicating some differences may exist between individual and team sport athletes. In truth, training variables and coaching strategies are much easier to individualize for individual sport athletes, which could lead to certain types of athletes selecting certain sports.

#### 4.11.3. Practical Implications of Motivation

Vallerand [163] suggested the most positive performance outcomes are derived from intrinsic motivational means, whereas lesser or negative outcomes may be associated with extrinsic means (external and introjected regulation) and amotivation. As coaches, it is important to consider the three basic needs of the SDT as well as the situational and contextual determinants when designing and implementing programs. An example that may impact an athlete’s motivation negatively could include programming an exercise that is too complex for the athlete’s present skill level. This would highlight incompetence of the athlete by a situational determinant of failure, resulting in a contextual determinant that lowers motivation. Another example is improperly pairing two athletes of differing ability together at a squat rack during a busy weight room training session. This would create relatedness and competence issues by introducing a situational determinant of competition, with the contextual determinant of teammates observing the competence deficit also negatively affecting the motivational climate. Analyzing the third component of SDT, some amount of autonomy can be included within programing as well. Coaches may select a variety of places within a program and allow athletes ownership of certain elements. For example, athletes could choose between two similar abdominal workout sequences at the end of a team training session. This choice, while of potentially limited impact to the overall training program, can foster feelings of autonomy among athletes, impacting the athletes’ intrinsic regulated motivation. 

Goal setting can be an important element of the athlete experience—it is achievement of goals that gives meaning to the work investment put forth to achieve them [178]. Within AGT, goals can range in scope from very specific to global goals [179]. Specific goals include goals focused on tasks directed at skills and skill mastery, which tend to lead to better motivation, participant investment, improved persistence, higher performance, greater satisfaction and enjoyment, and more positive feelings about self and the task [178]. Global goals drive the “why” of doing something [179]. These may be realized through the creation of a vision, mission, values, and standards system that can also develop alignment within the group or team [180]. 

The environment (contextual determinant) that is created by the coach should be an intentional aspect of the program design process due to its impact on motivation. Coaches should strive to create an environment that allows athletes to grow in competency, to feel connected to teammates and the coaching staff, and includes some element in training that fosters satisfaction, which is important to sustained involvement in physical development, particularly for younger athletes [181]. It is important to note that the leadership style of the coach can impact the environment created. A servant leadership style that is more task (vs. outcome) oriented has been shown to improve intrinsic motivation and may lead to greater athlete satisfaction [182]. 

Motivation awareness is another critical influence upon athlete motivation that may be manipulated by a coach. Assessments may be applied to investigate athlete levels of intrinsic motivation, extrinsic motivation, or amotivation. By understanding the type and current level of motivation and what drives each individual athlete, coaches can adapt their behaviors, the climate, and goals—all of which can help improve motivation [183]. Coaches seeking to explore athletes’ intrinsic and extrinsic motivation may consider the Sport Motivation Scale—II [184], the Exercise Motivation Scale [185], or the Motivation for Physical Activity Measure—Revised [186]. 

*Actionable points:* Coaches should strive to create an environment that optimizes motivation, fosters athlete satisfaction, and allows for strategic goals to be set by the athlete and coach. Coaches may optimize strategies after obtaining knowledge about athletes’ motivation type.

## 5. Conclusions

Clearly nonresponding athletes should be investigated by SC coaches, sport coaches, and sport support specialists. Data from testing sessions and monitoring processes may be used to classify nonresponders following use of appropriate statistical analysis and over multiple data collection sessions. SC coaches must use caution and diligence in classifying an athlete as a nonresponder, as many factors may explain a lack of improvement. Variation in performance and measurement error may be substantial, the magnitude of which may depend on the variable(s) measured. Attempting to optimize training is a complex issue, and coaches face many practical challenges in the effort to keep training and life stressors in balance with training goals [45,87]. As a result of these challenges, establishing collective priorities in training based on holistic conversations among the sport coaches and specialist support team may have tangible value to the athlete. As they investigate outcomes of training and processes that contribute to improved performance, SC coaches and other stakeholders may observe issues within sport organizations and provide feedback to administrators and senior coaches in order to help provide solutions [25,45]. Committing to an athlete-centric training model informed by a monitoring program is likely to result in the improved awareness of coaches and superior outcomes for athletes. Athletes require education on nutrition, hydration, sleep, alcohol and illicit drugs, as well as strategies that may improve coping skills. Coaches should consider employing athlete-specific strategies in order to optimize motivation.

## Figures and Tables

**Table 1:** Sleep improvement recommendations.

Frequently Recommended Strategies *
Acquire at least 8 h of sleep per day; up to 10–12 h in training periods
Use brief naps (30 min) after lunch
Establish a regular pre-sleep routine and habits.
Use relaxation, goal-setting, imagery, and positive self-talk to combat anxiety
Employ appropriate recovery strategies after training
Avoid watching television or using electronic devices in bed
Avoid caffeine later in the day
Ensure room is cool, quiet, and dark
Employ relaxation strategies as you settle down in bed

* Adapted from [103,105].

## Data Availability

Not applicable.
